# ^18^F-FDG PET/CT for Response Assessment in Pediatric Sebaceous Carcinoma of the Parotid Gland

**DOI:** 10.3390/diagnostics10110908

**Published:** 2020-11-06

**Authors:** Adrien Holzgreve, Thomas Pfluger, Irene Schmid, Orlando Guntinas-Lichius, Wolfgang G. Kunz, Jens Ricke, Peter Bartenstein, Nathalie L. Albert, Marcus Unterrainer

**Affiliations:** 1 Department of Nuclear Medicine, University Hospital, LMU Munich, 81377 Munich, Germany; Thomas.Pfluger@med.uni-muenchen.de (T.P.); Peter.Bartenstein@med.uni-muenchen.de (P.B.); Nathalie.Albert@med.uni-muenchen.de (N.L.A.); 2 Department of Pediatrics, Dr. von Hauner Children’s Hospital, University Hospital, LMU Munich, 80337 Munich, Germany; Irene.Schmid@med.uni-muenchen.de; 3 Department of Otorhinolaryngology, Jena University Hospital, 07747 Jena, Germany; Orlando.Guntinas@med.uni-jena.de; 4 Department of Radiology, University Hospital, LMU Munich, 81377 Munich, Germany; Wolfgang.Kunz@med.uni-muenchen.de (W.G.K.); Jens.Ricke@med.uni-muenchen.de (J.R.); Marcus.Unterrainer@med.uni-muenchen.de (M.U.)

**Keywords:** ^18^F-FDG PET/CT, pediatric sebaceous salivary gland carcinoma, meibomian tarsal gland carcinoma, parotid gland, therapy monitoring, ORPHA 276145

## Abstract

A 16-year-old male patient underwent ^18^F-FDG PET/CT staging after multiple surgical resections and radiotherapy of an uncommon metastatic pediatric sebaceous carcinoma of the parotid gland. Initial PET/CT imaging exhibited a recurrent paravertebral metastasis (C4) as well as a metabolically active tumor tissue at the primary site. Follow-up PET/CT after radiotherapy of the cervical spine (C4) and four cycles of chemotherapy with cisplatin and palbociclib revealed complete functional remission in the cervical spine and partial remission at the primary site. This case illustrates the ^18^F-FDG-uptake behavior and the disease course of a very rare malignant epithelial tumor of the salivary glands.

We present a 16-year-old male patient with recurrent metastatic sebaceous carcinoma in the left parotid gland. The tumor was diagnosed at the age of 9 years. Previous multimodal treatments between the age of 9 and 16 years included radical parotidectomy with partial resection of the left mandible, lower jaw and facial nerve reconstruction, repeated local re-resections and local adjuvant radiotherapy for local tumor recurrences, as well as spinal surgery for vertebral metastasis. Prior to further therapies, ^18^F-FDG PET/CT imaging with 232 MBq ^18^F-FDG and contrast-enhanced CT exhibited local re-recurrence in the left lower jaw with highly elevated glucose consumption (SUV_max_ 8.3; see Figure 1A) as well as an intra- and extraspinal recurrence of metastasis in the cervical spine (C4) with similarly elevated metabolism (SUV_max_ 8.8; see Figure 2A). Subsequently, a combined radiotherapy of C4 and chemotherapy with cisplatin and palbociclib, a cyclin-dependent kinase inhibitor, was initiated. The patient returned for follow-up after three months. Whereas no morphological changes were seen at the primary site (see red arrow in Figure 1), ^18^F-FDG PET imaging revealed a slightly decreased metabolic activity (SUV_max_ 7.1; see Figure 1B). The spinal metastasis showed a complete functional remission (see Figure 2B) on ^18^F-FDG PET.

Sebaceous carcinoma is an aggressive malignant tumor originating from the pilosebaceous unit, usually occurring in the periorbital area and less frequently in extraocular regions with an overall incidence of 1–2 per 1 million individuals per year [1,2,3]. Ectopic occurrence in the parotid gland, however, is a very rare condition that has only been reported in few cases [4,5]. The current patient is relatively young with an age of 9 years at initial diagnosis, whereas the median age at diagnosis of sebaceous carcinoma is 73 years [1,2]. Only very few cases of pediatric sebaceous carcinoma have been reported so far, but none of them with parotid location as in the current case [6,7,8]. An association of sebaceous carcinoma with Muir–Torre syndrome, a variant of Lynch syndrome (HNPCC), has been described [2,9]. First-line therapy generally consists of surgical removal [9]. Only a single case of ^18^F-FDG PET/CT imaging in a sebaceous carcinoma of the parotid gland in an adult patient has been reported so far, demonstrating the general feasibility of ^18^F-FDG PET/CT imaging in this tumor entity [10].

In clinical routine, sebaceous carcinoma has to be considered as a very rare differential diagnosis of a tumor in the parotid gland, even in young patients (ORPHA 276145). As illustrated by the current case, hybrid imaging using ^18^F-FDG PET/CT depicts changes of glucose consumption during therapy in sebaceous carcinoma, and may provide additional information for evaluation of therapy response beyond the morphological extent on CT. However, it still has to be clarified, whether assessment of glucose consumption in pediatric sebaceous carcinoma of the parotid gland improves clinical management and results in better patient outcomes. Due to the extreme rarity of the entity, prospective studies including PET/CT imaging are unlikely to be available; however the assumption of an added value seems plausible—e.g., when functional remission as in the current case indicates a therapy response—as a benefit of PET imaging in therapy management has likewise been shown for other conditions in pediatric oncology [11,12,13]. Hence, a mere unspecific, treatment-related uptake seems highly unlikely. Overall, hybrid imaging using PET/CT, but also PET/MRI should be considered for therapy monitoring in this entity, particularly in young patients.

## Figures and Tables

**Figure 1:**
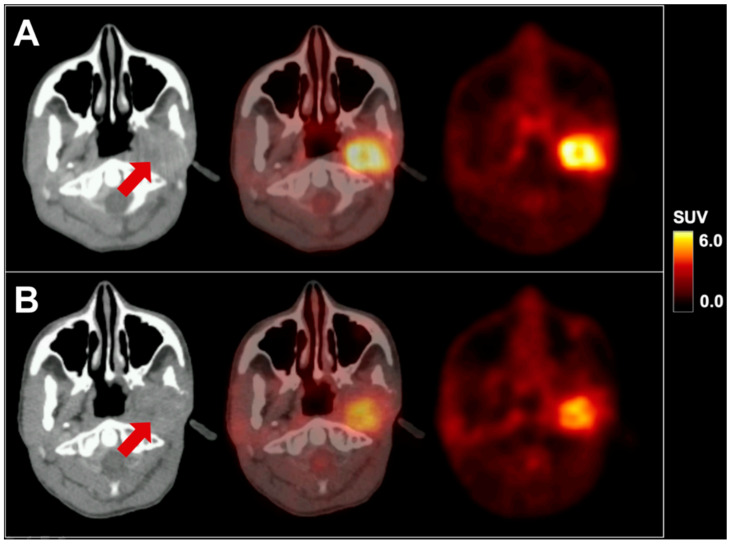
^18^F-FDG PET/CT in axial planes of a pediatric sebaceous carcinoma before (**A**) and after therapy (**B**). The red arrow indicates the primary site in the left parotid gland. SUV = standardized uptake value.

**Figure 2:**
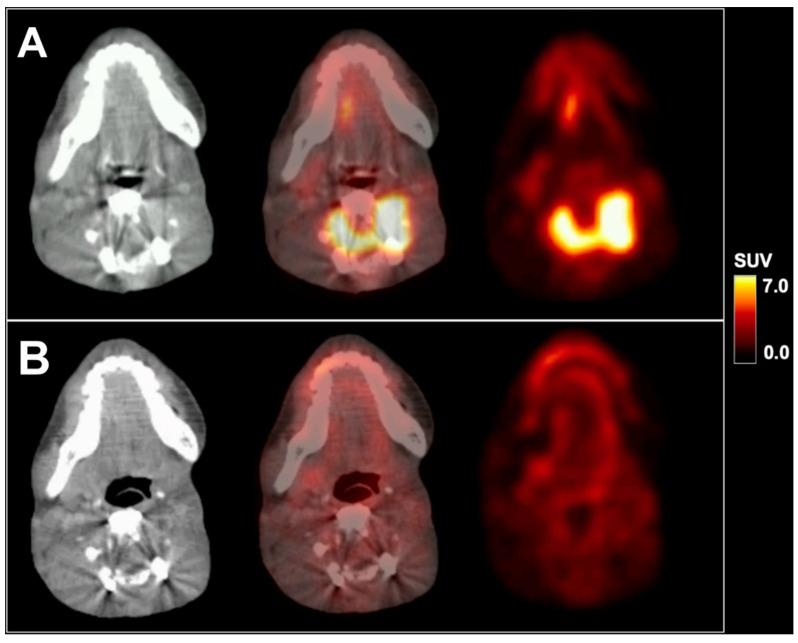
^18^F-FDG PET/CT in axial planes of a metastasis in the cervical spine before (**A**) and after radiochemotherapy (**B**). SUV = standardized uptake value.
